# Dead crow densities and human cases of West Nile virus, New York State, 2000.

**DOI:** 10.3201/eid0704.010411

**Published:** 2001

**Authors:** M Eidson, J Miller, L Kramer, B Cherry, Y Hagiwara, West Nile Virus Bird Mortality Analysis Group

**Affiliations:** Zoonoses Program, New York State Department of Health, Rm. 621 ESP Corning Tower, Albany, NY 12237, USA. mxe04@health.state.ny.us

## Abstract

In 2000, Staten Island, New York, reported 10 human West Nile virus cases and high densities of dead crows. Surrounding counties with <2 human cases had moderate dead crow densities, and upstate counties with no human cases had low dead crow densities. Monitoring such densities may be helpful because this factor may be determined without the delays associated with specimen collection and testing.


### Dead Crow Densities and Human Cases of West Nile Virus, New York State, 2000

**Millicent Eidson,* Jim Miller,† Laura Kramer,* Bryan Cherry,† Yoichiro Hagiwara,* and the West Nile Virus Bird Mortality Analysis Group[1]**

*New York State Department of Health, Albany, New York, USA; and †New York City Department of Health, New York City, New York, USA

In 2000, Staten Island, New York, reported 10 human West Nile virus cases and high densities of dead crows. Surrounding counties with ≤2 human cases had moderate dead crow densities, and upstate counties with no human cases had low dead crow densities. Monitoring such densities may be helpful because this factor may be determined without the delays associated with specimen collection and testing.

West Nile (WN) virus was first recognized as a cause of encephalitis in humans and other animals in the United States in 1999, and dead bird surveillance in the northeastern states provided a valuable window into the temporal and geographic distribution of viral activity (1). In 2000, a real-time web-based dead bird surveillance system established for New York State (NYS) (2) identified dead crow sightings and laboratory positive dead birds before the onset date for the first human WN virus cases (3). Viral activity appeared to be widely distributed in 2000, with WN virus-positive birds, mammals, or mosquitoes reported from the District of Columbia and 12 states, from New Hampshire to North Carolina (4). However, the 21 human WN virus cases, with a clinical spectrum from mild illness to fatal encephalitis, were limited to New York City (NYC), New Jersey, and Connecticut (5). We compared the number of human cases with dead bird surveillance factors by county in NYS in 2000 to assess possible temporal correlations.

### The Study

Fourteen human WN virus cases were confirmed from NYS in 2000, all from NYC (10 from Staten Island, 2 from Brooklyn, and 1 each from Queens and Manhattan) (4,5). A total of 1,263 WN virus-positive dead birds were reported from 61 of 62 NYS counties, including the five NYC boroughs (3). In NYS, 71,332 dead bird sightings were reported from all 62 counties; 17,571 (24.6%) were American Crows (3).

We examined the variability by county for dead bird surveillance factors for NYS in 2000 and report results for the density of dead crow sightings (calculated as the total number of sightings divided by the square-mile area of the county). Estimates of county land area were obtained from 1990 land area data; estimates of human population were obtained from 1999 estimates of the U.S. Census Bureau (6).

Address for correspondence: Millicent Eidson, Zoonoses Program, New York State Department of Health, 621 Corning Tower, Empire State Plaza, Albany, New York 12237, USA; fax: 518-473-6590; e-mail: mxe04@health.state.ny.us

Comparing the total number of human WN virus cases and the dead crow densities by county for 2000 (Figure 1) allows three groups of NYS counties to be distinguished: Staten Island (10 human cases and 33.3 dead crows per square mile), the other boroughs in NYC and surrounding counties that had WN virus activity both in 1999 and 2000 (≤2 human cases and 3-12 dead crows per square mile for each county), and upstate New York (no human cases and <1.0 dead crow per square mile).

For the four NYC boroughs with human WN virus cases, the weekly densities of dead crows were graphed with the dates of onset of human cases. In Staten Island (Figure 2A), a steep rise in the density of dead crows began 2 weeks before the onset of the first human case on July 20 (7), before laboratory confirmation of viral activity. The peak of 5.9 reported crows per square mile coincided with press announcements of a possible human WN virus case (later

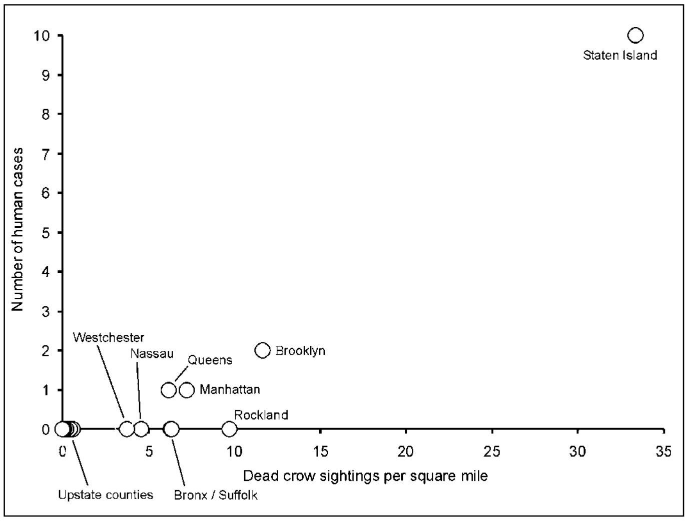

Figure 1. Annual dead crow density (number of dead crow sightings per square mile) compared with number of human cases, New York State, 2000.

[1]Madhu Anand, Rockland County Department of Health; Ada Huang, Westchester County Department of Health; Clare Bradley, Suffolk County Department of Health Services; Angela Pettinelli, Nassau County Department of Health; Kate Schmit, Barbara Wallace, Perry Smith, Geraldine Johnson, Dennis White, Division of Epidemiology, New York State Department of Health; and Kristen Bernard, Alan Dupuis, Gregory Ebel, Susan Jones, Elizabeth Kauffman, Joseph Maffei, Arbovirus Laboratory, New York State Department of Health.

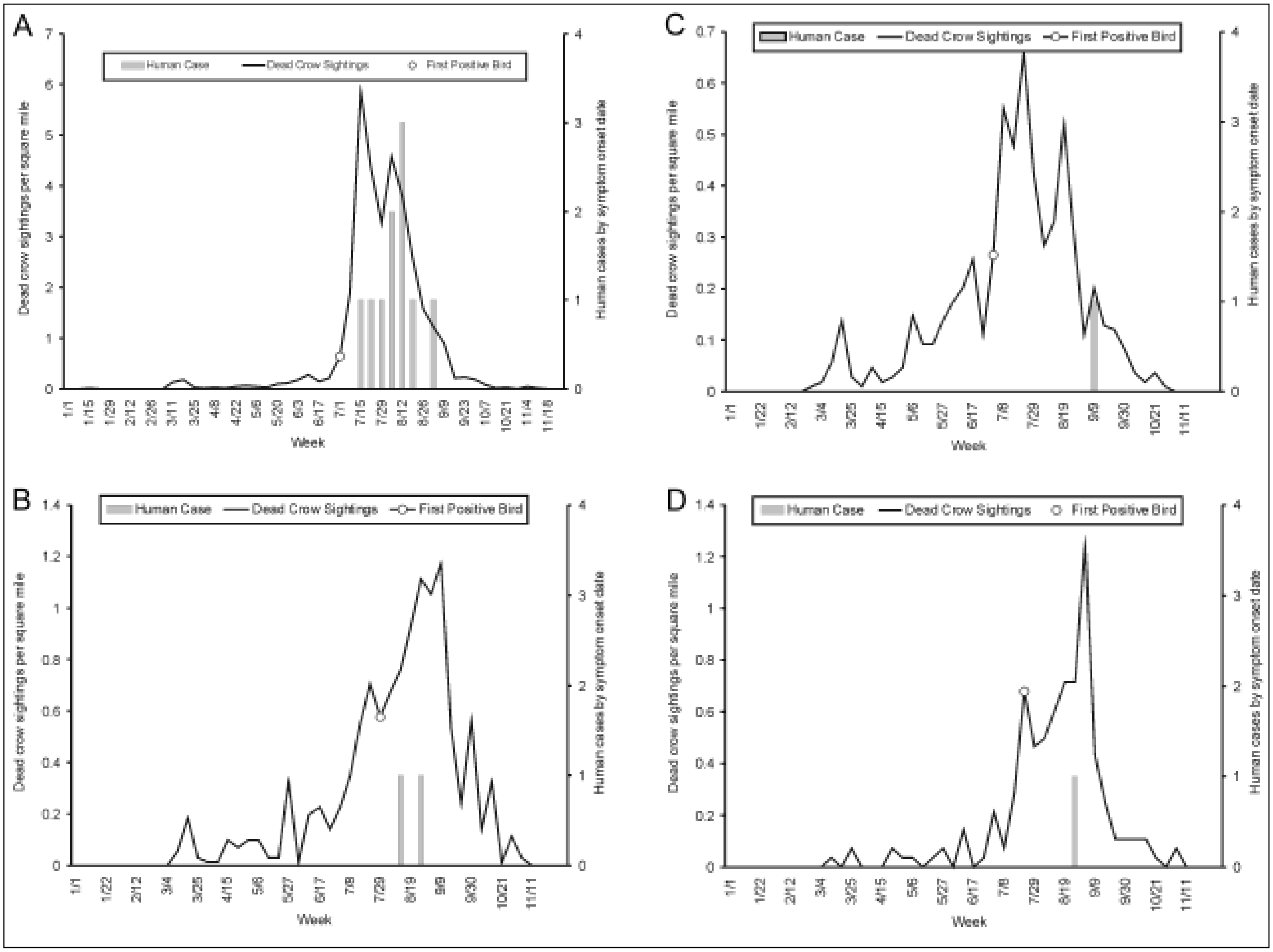

Figure 2. Dead crow density (number of dead crow sightings per square mile) compared with number of human cases, by week. A. Staten Island, axis scale for weekly dead crow density 0 to 7; B. Brooklyn, axis scale for weekly dead crow density 0 to 1.4; C. Queens, axis scale for weekly dead crow density 0 to 0.7; D. Manhattan, axis scale for weekly dead crow density 0 to 1.4.

determined to be negative for WN virus) and the first WN virus-positive crow (collected 2 weeks earlier). In the other three NYC boroughs with one or two human cases, WN virus-positive birds (American Crows in Queens and Manhattan and a Fish Crow in Brooklyn) were found, and dead crow densities increased before the dates of onset of human case (Figures 2B-D), with a maximum weekly dead crow density in Manhattan of 1.25 the week after the date of onset of the human case (Figure 2D).

The rest of the area with WN virus activity both in 1999 and 2000—the Bronx, the two counties immediately north of NYC (Westchester and Rockland), and the two counties to the east (Nassau and Suffolk)—did not have human WN virus cases in 2000, and the weekly dead crow densities never exceeded 1.0. Of the upstate NYS counties with evidence of viral activity only in 2000, none exceeded 0.1 dead crow sightings per square mile per week.

### Conclusions

Overall in 2000 and on a weekly basis, three levels of dead crow densities were identified, with high levels in Staten Island, moderate levels in surrounding counties that also had viral activity in 1999, and low levels in upstate counties. Staten Island also had the highest number of human cases, while few human cases were reported from the other surrounding areas with viral activity in 1999 and 2000, and none were reported from upstate counties. This pattern was supported by data from Connecticut showing moderate dead crow densities in a county that had viral activity in both 1999 and 2000 and one WN virus-positive person with a mild illness in 2000 (8). Similarly, Staten Island had a higher proportion of birds that tested positive and higher mosquito infection rates (9,10).

These and other analyses of WN virus in the northeastern United States in 2000 (3,8-10) indicate that dead bird and mosquito surveillance can be useful for monitoring viral activity and the potential for human cases in this geographic area. Tracking dead crow density avoids delays inherent in specimen collection and testing and thus may be more helpful on a weekly basis to permit rapid recognition of trends in viral activity and the potential for occasional human cases or an outbreak.

Whether dead crow densities will be associated with the number of human cases in future years or other geographic areas is unknown. If an area has few crows, crows become immune, or dead crow reporting is inadequate or delayed, an increase in dead crow densities may not be observed before the onset of human cases. Development of spatial statistical procedures to quickly detect geographic clusters of dead crow sightings may be valuable for identification of high-risk areas that cross geopolitical boundaries such as states, counties, or towns.

### Acknowledgments

The authors thank Ward Stone and the Wildlife Pathology Unit, New York State Department of Environmental Conservation; Scott Campbell, Catherine D'Aleo, Stacey Gavin, David Graham, Brian Hunderfund, Mike Luke, Karin Rhines, and local and state agencies that provided critical dead bird surveillance data; and Bryon Backenson, Hwa-Gan Chang, Ivan Gotham, Dale Morse, John Napoli, Kiet Ngo, Kristine Smith, Amy Willsey, and Donna Young for assistance with the West Nile virus dead bird surveillance system.

Dr. Eidson is State Public Health Veterinarian and Director of the Zoonoses Program, New York State Department of Health. In addition, she is an associate professor in the Department of Epidemiology, State University of New York School of Public Health, and a diplomate of the American College of Veterinary Preventive Medicine. Her research focuses on rabies and West Nile virus.

### References

1. Eidson M, Komar N, Sorhage F, Nelson R, Talbot T, Mostashari F, et al. Crow deaths as a sentinel surveillance system for West Nile virus in the Northeastern United States, 1999. Emerg Infect Dis 2001:7:615-20.
2. Gotham IJ, Eidson M, White DJ, Wallace BJ, Chang HG, Johnson GS, et al. West Nile virus: A case study in how New York State health information infrastructure facilitates preparation and response to disease outbreaks. Journal of Public Health Management and Practice 2001;7(5):79-89.
3. Eidson M, Kramer L, Stone W, Hagiwara Y, Schmit K, and the New York State West Nile Virus Avian Surveillance Team. Dead bird surveillance as an early warning system for West Nile virus. Emerg Infect Dis 2001;7:631-5 .
4. Centers for Disease Control and Prevention. Update: West Nile virus activity—Eastern United States, 2000. MMWR Morb Mortal Wkly Rep 2000;49:1044-7.
5. Centers for Disease Control and Prevention. Serosurveys for West Nile Virus infection—New York and Connecticut counties, 2000. MMWR Morb Mortal Wkly Rep 2001;50:37-9.
6. United States Census Bureau, State and County QuickFacts. Available at: URL: http://quickfacts.census.gov/qfd/.
7. Centers for Disease Control and Prevention. Update: West Nile virus activity—Northeastern United States, January-August 7, 2000. MMWR Morb Mortal Wkly Rep 2000:49;714-7.
8. Hadler J, Nelson R, McCarthy T, Andreadis T, Lis MJ, French R, et al. West Nile virus surveillance in Connecticut in 2000: An intense epizootic without high risk for severe human disease. Emerg Infect Dis 2001;7:636-42.
9. White DJ, Kramer LD, Backenson PB, Lukacik G, Johnson G, Oliver J, et al. Mosquito surveillance and polymerase chain reaction detection of West Nile virus, New York State. Emerg Infect Dis 2001;7:643-9.
10. Bernard KA, Maffei JG, Jones SA, Kauffman EB, Ebel GD, Dupuis AP, et al. West Nile virus infection in birds and mosquitoes, New York State, 2000. Emerg Infect Dis 2001;7:679-85.
